# An individualized nomogram for predicting and validating spread through air space (STAS) in surgically resected lung adenocarcinoma: a single center retrospective analysis

**DOI:** 10.1186/s13019-023-02458-0

**Published:** 2023-11-21

**Authors:** Jing Wang, Yuanshan Yao, Dongfang Tang, Wen Gao

**Affiliations:** Department of Thoracic Surgery, Shanghai Key Laboratory of Clinical Geriatric Medicine, Huadong Hospital Affiliated to Fudan University, Shanghai, 200041 China

**Keywords:** Surgically resected lung adenocarcinoma, STAS, Nomogram, Prediction

## Abstract

**Objective:**

A single-center study was conducted to explore the association between STAS and other clinical features in surgically resected adenocarcinoma to enhance our current understanding of STAS.

**Methods:**

We retrospectively enrolled patients with lung adenocarcinoma (n = 241) who underwent curative surgeries. Patients undergoing surgery in 2019 were attributed to the training group (n = 188) and those undergoing surgery in January 2022 to June 2022 were attributed to the validation (n = 53) group. Univariate and multivariate logistic regression analyses were used to identify predictive factors for STAS, which were used to construct a simple nomogram. Furthermore, ROC and calibration curves were used to evaluate the performance of the nomogram. In addition, we conducted decision curve analysis (DCA) to assess the clinical utility of this nomogram.

**Results:**

In our cohort, 52 patients were identified as STAS-positive (21.6%). In univariate analysis, STAS was significantly associated with age, surgical approach, CEA, CTR (Consolidation Tumor Ratio), TNM stage, tumor grade, gross tumor size, resection margin, vessel cancer embolus, pleural invasion, lymph node metastasis, high ki67 and positive PD-L1 staining (P < 0.05). Lower age, CTR > 0.75, vessel cancer embolus, high Ki67 and PD-L1 stain positive were significant predictors for STAS during multivariate logistics analysis. A simple nomogram was successfully constructed based on these five predictors. The AUC values of our nomogram for the probability of tumor STAS were 0.860 in the training group and 0.919 in the validation group. In addition, the calibration curve and DCA validated the good performance of this model.

**Conclusion:**

A nomogram was successfully constructed to identify the presence of STAS in surgically resected lung adenocarcinoma patients.

## Introduction

Lung cancer remains the leading cause of mortality among all malignancies worldwide. Invasion and metastasis are the main factors that determine the prognosis of cancer patients. Lymphatic, vascular and pleural invasions are the main mechanisms underlying lung cancer invasion and metastasis. In 1980, Tamai et al. first reported spread through air space (STAS) as isolated or a cluster of tumor cells floating in the alveoli [1]. In 2015, the World Health Organization introduced the concept of spread through air space as a fourth potential invasive pattern [2]. STAS was defined as tumor cell clusters found beyond the main body of the tumor in at least one alveolar space [3]. Blaauwgeers and his colleagues questioned the presence of STAS and denied the effectiveness of STAS as a novel invasive pattern. They perceived these loose cancer cell clusters as derived from intraoperative extrusion by thoracic surgeons or postoperative pathology slices from contamination during processing by pathologists [4]. However, no consensus has been reached up to now. Recently, Metovic et al. investigated 51 NSCLC surgical specimens, including 35 lung adenocarcinoma and suggested STAS was an existing biological phenomenon rather than induced by surgeons or pathologists [5]. STAS has been associated with various types of lung cancer specimens including adenocarcinoma, squamous cell carcinoma, small cell lung cancer and so on [6, 7], but is reportedly more common in lung adenocarcinoma patients, accounting for 70% of all non-small cell lung cancer cases [8].

Herein, we comprehensively collected the basic clinicopathological features, combined with some easily accessible hematological indicators, gene mutational features and PD-L1 expression level, which were obtained from the medical records system of our institution. Then univariate and multivariate logistic regression were used to probe some independent clinical parameters associated with STAS. Finally, a nomogram based on the above parameters was developed and validated. We hope our established nomogram can provide a reference for clinical application.

## Materials and methods

### Patient selections

**Fig. 1:**
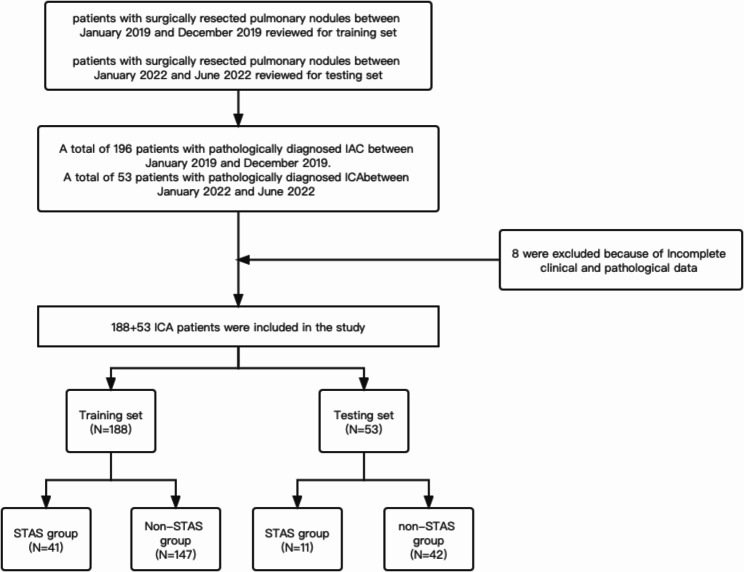
Flowchart for patient selection

In our study,188 patients accepting surgery in 2019 were included as the training group, and 53 patients accepting surgery from January 2022 to June 2022 were classified as the validation group. Lung adenocarcinoma patients who underwent lobectomy, wedge resection, segmentectomy and pneumonectomy with en bloc mediastinal lymph node dissection were included in this study. Patients were excluded for the following reasons: (1) adenocarcinoma in situ or minimally invasive adenocarcinoma on postoperative pathology; (2) histopathological diagnosis of invasive mucinous adenocarcinoma; (3) Treatment with neoadjuvant therapy before surgery; (4) Presence of other primary malignancies; (5) Patients with incomplete medical records. The flow chart for patient selection is shown in Fig. 1. The study was approved by the ethics committee of Huadong Hospital, and no informed consent was obtained from patients due to the retrospective nature of our study.

### Methods

#### Pathologic STAS evaluation

**Fig. 2:**
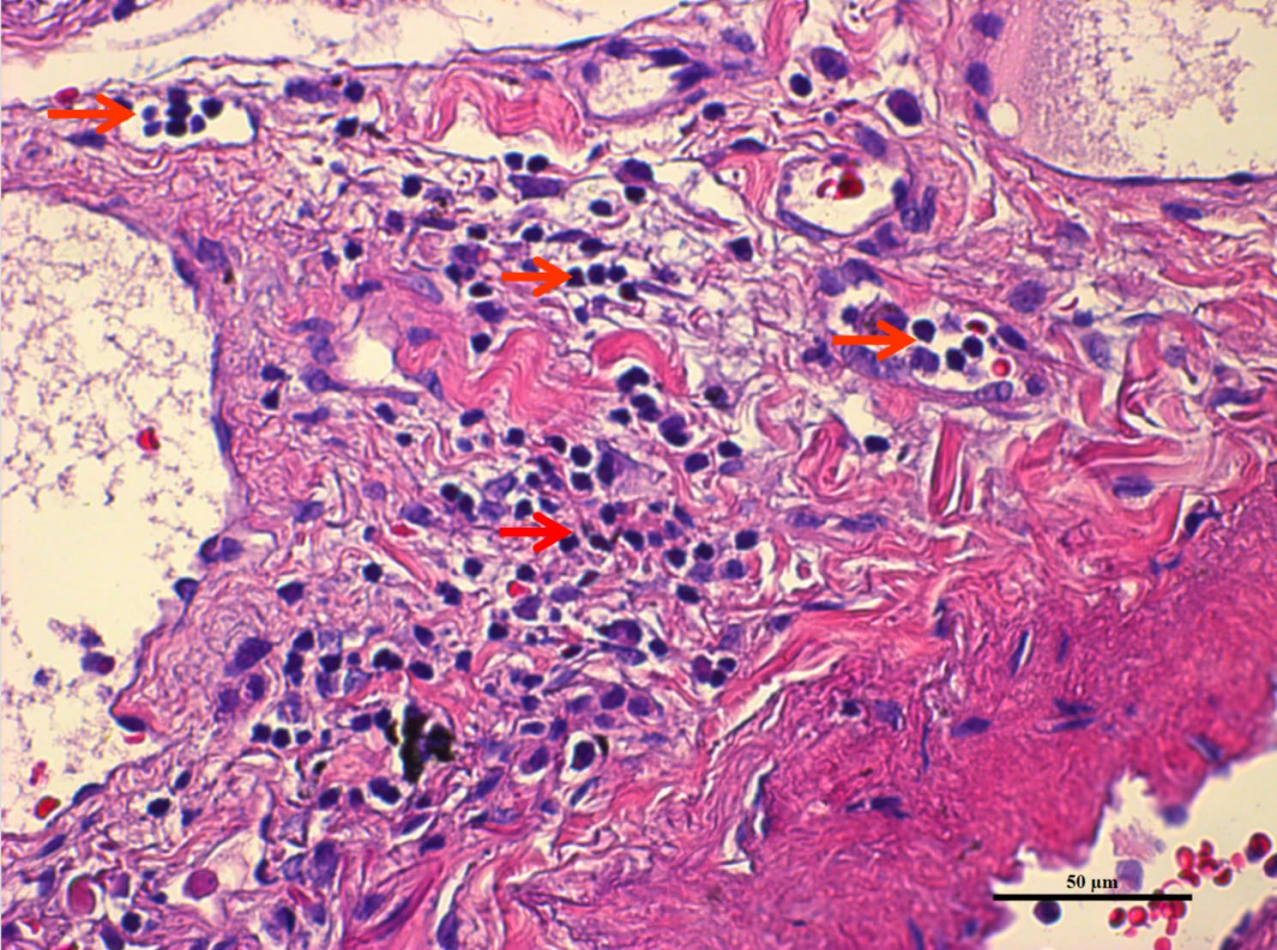
Representative pathological images of STAS(Red arrow indicated the tumor nest)

Hematoxylin and eosin histological staining was conducted to assess STAS status in resected adenocarcinoma tissues. Briefly, the tissues were embedded in paraffin and fixed with formalin. Tumor STAS implies that tumor cells are found in the normal pulmonary tissues outside the boundary of the main tumor, according to the literature [6, 9, 10]. It is generally accepted that the micropapillary subtype, solid nets subtype and single cancer cell subtype are predominantly found in STAS [11]. To differentiate “blade contamination” from real STAS, the following cases were excluded: (1) scattered tumor cells caused by the blade during specimen processing; (2) individual isolated tumor cells detected far away from the main tumor without disseminating in a continuous form [4, 5, 12]. Two senior pathologists independently reviewed all the slides, and a consensus-based decision was made for points of disagreement. The representative pathological images of STAS are listed in Fig. 2.

#### Immunohistochemistry (IHC)

Immunohistochemistry was used to detect PD-L1 and PD-1 expression levels in adenocarcinoma tissues. Briefly, the tissues were embedded in paraffin and then fixed with formalin. Then the tissues (4–5 μm-thick) were incubated overnight at 4 °C with rabbit anti-PD-L1 monoclonal antibody (E1L3N, 1:100, CST) and rabbit anti-PD-1 monoclonal antibody (MX033,1:100, MaiXin). Immunostaining was performed using the EnVision + System-HRP (AEC) (K4005, Dako, Glostrup, Denmark). Positive PD-L1 and PD1 expression were observed in the membrane. The expression level was classified into two groups: < 1 was grouped as negative, while ≥ 1% was grouped as positive [13].

#### Gene mutational analysis

The mutational status of targetable oncogenic driver gene EGFR and ALK was assessed through the standard polymerase chain reaction based method [14]. Then ALK fusion was confirmed by the fluorescence in-situ hybridization method (FISH). Rare mutation sites and common mutation sites of EGFR were all recorded in our cohort.

#### Other clinicopathological indexes

Age, gender, location of the lesions, surgical approach, tumor differentiation grade, tumor margin, pleural invasion, tumor size, pTNM stage, Ki67, intravascular cancer embolus, NLR (neutrophil to lymphocyte ratio), PLR (platelet to lymphocyte ratio) and several preoperative blood indexes were all collected from electronic medical records. The pTNM stage was stratified based on the 8th edition of New TNM staging in lung cancer [15]. Positive pleural invasion was defined as tumor invasion beyond the elastic lamina of the pulmonary tissue. The histological types of lung adenocarcinoma mainly included lepidic, papillary, acinar, micropapillary, and solid types. The threshold for predominant histological type was set to 20%. According to the published literature, poorly differentiated types included micropapillary or solid type ≥ 20%; moderately differentiated types included acinar or papillary type, and well-differentiated types included the lepidic type without micropapillary or solid type ≥ 20% [16].

### Statistical methods

All patient databases were established, and data analysis was performed using SPSS 22.0 statistical software and R version 4.0.2. The binary logistic regression model was used for univariate analysis in the training group. Indexes with P < 0.05 were selected for multivariate logistic regression analysis. The independent predictive factors with P < 0.05 were then selected to establish a nomogram.

ROC and calibration curves were used to evaluate nomogram performance in both training and validation groups. In addition, a DCA analysis was conducted to assess the clinical usefulness of the nomogram.

## Results

### Patient characteristics

21.6% (n = 52) of patients were STAS-positive on HE staining. According to the satatistics from the Department of Pathology, five cases were categorized as true artifacts which were distinguished from STAS. These cases shared the same characteristics: loose tissue fragments were seen in blocks after cutting through tumor bed [17].The mean age of included patients was 63.8 [8.8] years (Range 35–83 years). Postoperative pathological diagnosis showed that most cases were early-stage lung cancer (I + II) (n = 218, 90.5%). The baseline characteristics of participants are listed in Table 1.

**Table 1:** Baseline characteristics of participants

Characteristics		Training group	Validation group	P Value
No. of cases		188	53	
Period of diagnosis		2019.1 ~ 2019.12	2022.1 ~ 2022.6	
Age	≤ 65 y	109 (58)	28 (52.8)	0.504
	＞65y	79 (42)	25 (47.2)	
Sex	Male	91 (48.4)	26 (49.1)	0.933
	Female	97 (51.6)	27 (50.9)	
Tumor site	Inferior left	32(17)	8(15.1)	0.722
	Superior left	47(25)	10(18.9)	
	Inferior right	36(19.1)	14(26.4)	
	Middle right	11(5.9)	4(7.5)	
	Superior right	62(33)	17(32.1)	
Surgical approach	Lobectomy	106(56.4)	25(47.2)	0.234
	Sublobar resection	82(43.6)	28(52.8)	
CEA	＞5	39(20.7)	8(15.1)	0.359
	≤ 5	149(79.3)	45(84.9)	
NSE	＞17	8(4.3)	9(17)	0.004
	≤ 17	180(95.7)	44(83)	
Cyfra21-1	＞3.3	58(30.9)	11(20.8)	0.151
	≤ 3.3	130(69.1)	42(79.2)	
CA199	＞34	7(3.7)	1(1.9)	0.822
	≤ 34	181(96.3)	52(98.1)	
CA724	＞8.2	13(6.9)	10(18.9)	0.009
	≤ 8.2	175(93.1)	42(81.1)	
CA125	＞35	8(4.3)	2(3.8)	1.000
	≤ 35	180(95.7)	51(96.2)	
NLR		2.22(1.68,2.92)	2.36 (1.81,3.42)	0.102
PLR		131.64 (105.30,164.36)	139.61 (111.65,169.08)	0.229
CT size		16 (12,25)	16 (12,23.5)	0.579
CTR	＞75%	90(47.9)	14(26.4)	0.013
	50%~75%	22(11.7)	14(26.4)	
	25%~50%	34(18.1)	11(20.8)	
	≤ 25%	42(22.3)	14(26.4)	
TNM stage	I + II	168(79.4)	50(94.3)	0.276
	III + IV	20(20.6)	3(5.7)	
STAS	+	41(21.8)	11(20.8)	0.869
	-	147(20.8)	42(79.2)	

### Association analysis in the training group

STAS was significantly associated with age, surgical approach, CEA, CTR, TNM stage, tumor grade, gross tumor size, resection margin, vessel cancer embolus, pleural invasion, lymph node metastasis, high ki-67 and positive PD-L1 stainnig (P < 0.05) (Tables 2-3).

**Table 2:** Correlation between the presence of STAS and clinical features

Patient characteristics		STAS		P value
		Present N = 41	Absent N = 147	
Age	＞65	9(22)	70(47.6)	0.003
	≤ 65	32(78)	77(52.4)	
Sex	Female	17(41.5)	80(54.4)	0.142
	Male	24(58.5)	67(45.6)	
Tumor site	Inferior left	10(24.4)	22(15)	0.436
	Superior left	7(17.1)	40(27.2)	
	Inferior right	9(22)	27(18.4)	
	Middle right	3(7.3)	8(5.4)	
	Superior right	12(29.3)	50(34)	
Surgical approach	Lobectomy	31(75.6)	75(51)	0.005
	Sublobectomy	10(24.4)	72(49)	
CEA	＞5	13(31.7)	26(17.7)	0.050
	≤ 5	28(68.3)	121(82.3)	
NSE	＞17	4(9.8)	4(2.7)	0.125
	≤ 17	37(90.2)	143(97.3)	
Cyfra21-1	＞3.3	13(31.7)	45(30.6)	0.893
	≤ 3.3	28(68.3)	102(69.4)	
CA199	＞34	2(4.9)	5(3.4)	1.000
	≤ 34	39(95.1)	142(96.6)	
CA724	＞8.2	1(2.4)	12(8.2)	0.353
	≤ 8.2	40(97.6)	135(91.8)	
CA125	＞35	3(7.3)	5(3.4)	0.509
	≤ 35	38(92.7)	142(96.6)	
NLR		2.34(1.71,3.17)	2.21(1.67,2.82)	0.655
PLR		109.46(135.47,170.67)	104.49(131.53,162.59)	0.759
CT size		20(12,28)	16(12,24)	0.179
CTR	≤ 25%	1(2.4)	41(27.9)	＜0.001
	25%~50%	4(9.8)	30(20.4)	
	50%~75%	3(7.3)	19(12.9)	
	＞75%	33(80.5)	57(38.8)	

**Table 3:** Correlation between the presence of STAS and pathological factors

		STAS		P value
		Present N = 41	Absent N = 147	
TNM stage	III + IV	11(26.8)	9(6.1)	＜0.001
	I + II	30(73.2)	138(93.9)	
Gross size		20(13,30)	15(12,24)	0.018
IASLC	3	25(61)	38(25.9)	＜0.001
	1,2	16(39)	109(74.1)	
margin	+	5(12.2)	4(2.7)	0.036
	-	36(87.8)	143(97.3)	
Vessel invasion	+	13(31.7)	6(4.1)	＜0.001
	-	28(68.3)	141(95.9)	
Pleural invasion	+	21(51.2)	46(31.3)	0.018
	-	20(48.8)	101(68.7)	
Lymph nodes	+	13(31.7)	13(8.8)	＜0.001
	-	28(68.3)	134(91.2)	
Ki67		30(10,45)	5(4,10)	＜0.001
EGFR	+	23(56.1)	83(56.5)	0.967
	-	18(43.9)	64(43.5)	
ALK	+	2(4.9)	2(1.4)	0.208
	-	39(95.1)	145(98.6)	
PD1	+	0(0)	2(1.4)	1.000
	-	41(100)	145(98.6)	
PDL1	+	20(48.8)	29(19.7)	＜0.001
	-	21(51.2)	118(80.3)	

### Multivariate logistic regression analysis in the training group

Multivariate logistic regression analysis demonstrated that lower age, CTR > 0.75, vessel cancer embolus, high Ki-67 and positive PD-L1 staining were independent predictive factors of STAS (Table 4).

**Table 4:** Multivariate logistic regression analysis of clinicopathological features for predicting STAS

	Beta coefficient	OR	95%CI	P value
Age	-1.639	0.194	0.073,0.52	0.001
Surgical approach	0.61	1.84	0.7,4.838	0.216
CTR				0.027
＞75%	2.551	12.825	1.564,105.201	
50%~75%	1.183	3.263	0.289,36.837	
25%~50%	1.613	5.016	0.508,49.511	
≤ 25%		1	…	
TNM stage	-0.539	0.583	0.073,4.669	0.612
Gross size	-0.005	0.995	0.964,1.027	0.762
IASLC	0.137	1.147	0.422,3.115	0.788
margin	1.542	4.673	0.635,34.386	0.13
Vessel invasion	1.471	4.354	1.299,14.593	0.017
Pleural invasion	-0.234	0.791	0.299,2.095	0.637
Lymph nodes	1.248	3.484	0.979,12.399	0.054
ki67	0.021	1.022	1.001,1.043	0.042
PDL1	1.191	3.291	1.309,8.274	0.011

### Construction of the nomogram

**Fig. 3:**
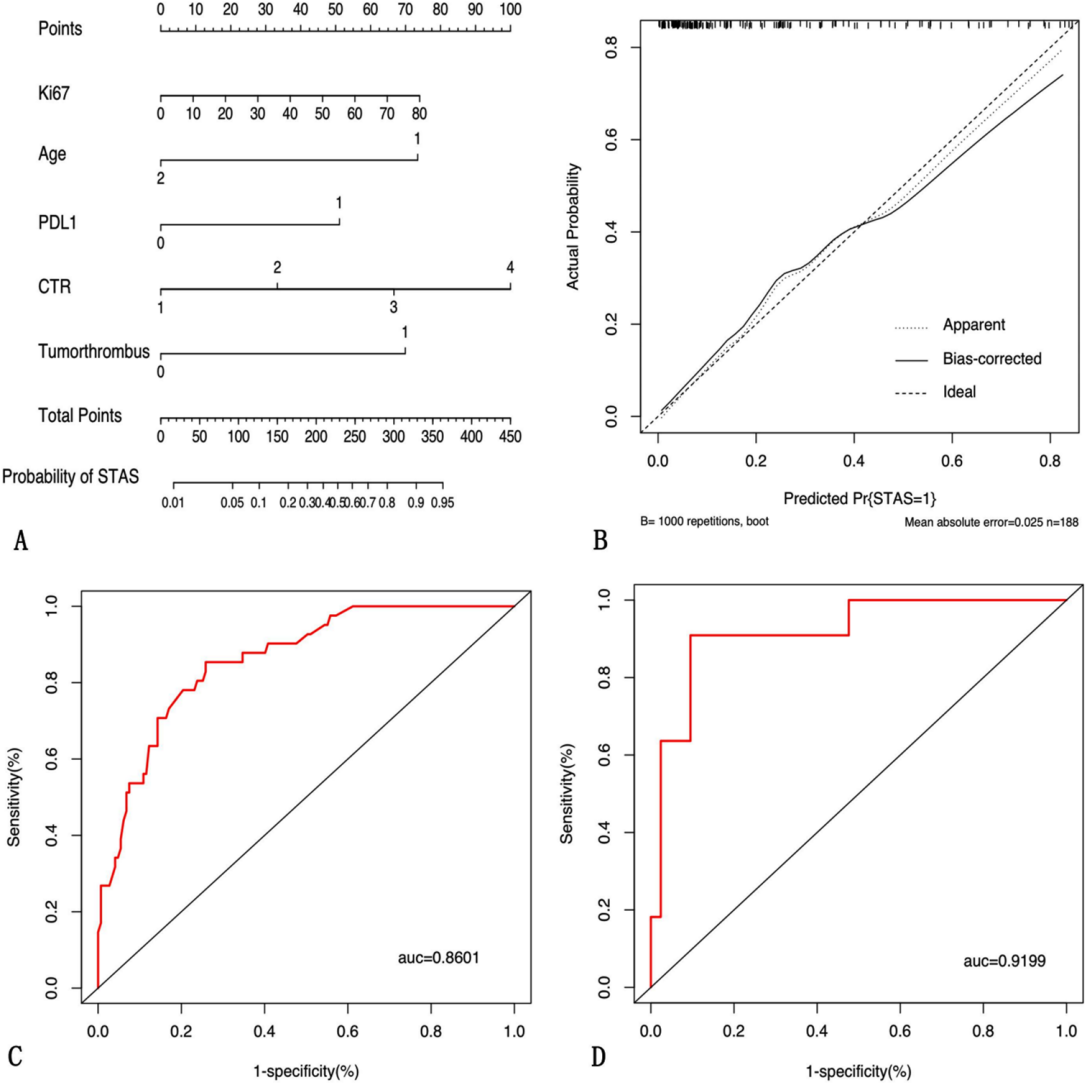
Nomogram for predicting the presence of STAS in adenocarcinoma patients. (**A**) The model for predicting the incidence of STAS. (**B**) Calibration curves of the nomogram to predict the presence of STAS in the training group. (**C-D**) The ROC curve of nomogram in the training cohort and validation cohort

A simple nomogram was established according to the multivariate logistic regression analysis (Fig. 3A). Age was a protective factor for the presence of STAS, while the other four indicators were risk factors for the presence of STAS.

### Model performance in the training group and validation group

**Fig. 4:**
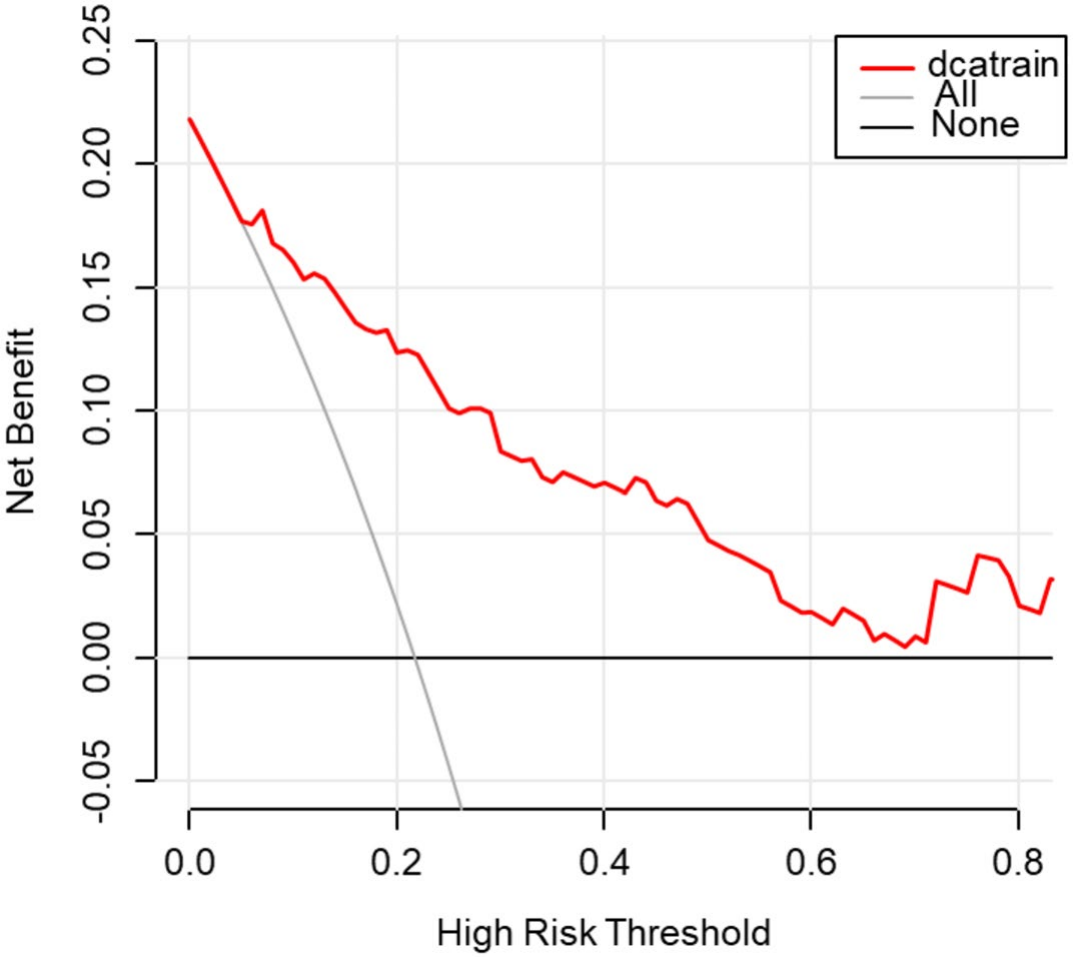
DCA analysis of the nomogram for predicting STAS in the training group

The calibration plot of the model displayed good agreement between the predicted and actual outcomes through bootstrapping validation method in the training group(Fig. 3B). Our nomogram yielded AUCs of 0.86 (95% CI 0.80–0.92) and 0.92 (95% CI 0.83–0.99) in the training group and validation group, respectively (Fig. 3C-D). In addition, DCA analysis showed a net benefit associated with using this model (Fig. 4).

## Discussion

At present, surgery remains the mainstay of treatment for early-stage lung cancer. With significant developments in high-resolution computed tomography achieved over the years, ground glass opacity lesions are increasingly detected. Sublobar resection is recommended for ground glass opacity lesions with less aggressive features [18]. However, a small proportion of patients developed locoregional recurrence after undergoing limited resection. In this respect, Gross and his colleagues investigated nine tumor tissues from resected lung adenocarcinoma patients who underwent additional lobectomy following wedge resection since STAS was detected in wedge resection specimens. They also confirmed the existence of STAS in additional resected lobectomy specimens and assumed that undetected STAS was a potential risk factor for postoperative recurrence [19]. Ogata et al. also reported that STAS was a prognostic predictor for stage I lung squamous cell cancer patients [20]. In recent years, several scholars have evaluated the potential of STAS as the fourth way of cancer dissemination. Nevertheless, STAS is an exclusion criterion, and much controversy surrounds the biological phenomena of STAS so far [11]. In addition, it remains unclear how to detect the presence of STAS in intraoperative specimens. Thus, we studied the incidence rate of STAS in resected lung adenocarcinoma patients at our single institution. Moreover, we explored several independent clinical parameters which were associated with STAS. Finally, a nomogram was developed to predict the occurrence of STAS.

Several studies have emphasized an association between STAS and clinicopathological indexes, many inconsistencies were reported. Our analysis showed that lower age, CTR > 0.75, vessel cancer embolus, high Ki-67 and positive PD-L1 staining were independent predictors for STAS in our cohort. Undoubtedly, most of these predictors can reflect greater tumor aggressiveness. Liu et al. demonstrated that CTR > 50% on CT imaging was associated with a high likelihood of STAS, suggesting lobectomy might be the best choice for patients with CTR > 50% [21]. Studies found that STAS is more prevalent in intravascular invasion lung adenocarcinoma, in accordance with our study [22, 23]. It is widely thought that tumor clusters which can easily migrate beyond the tumor edge are more likely to invade the structure of lymphatic or blood vessels from the viewpoint of tumor developmental biology. 3-D reconstruction images of STAS confirmed this complex biological behavior that STAS cells can spread to the normal alveolar walls via the vasculature [24]. PD-L1 expression is considered the most reliable biomarker to predict the outcome of immunotherapy at present [25]. So far, few studies have reported a definite association between STAS and PD-L1 expression level. Ge et al. recently analyzed 3075 NSCLC patients and found that PD-L1 expression level in tumor cells or stromal cells was significantly related to STAS in univariate analysis [26]. The exact mechanism underlying this phenomenon remains unknown. It is generally accepted that the expression of PD-L1, an important pathway for immune evasion, is regulated by the tumor microenvironment. Hence, we postulated that the tumor immune microenvironment might influence the occurrence of STAS. More studies are needed to reveal the detailed immune mechanism of STAS. Our study also substantiated that STAS was closely related to younger age, which was not depicted in the literature. We assumed that younger patients had greater metastatic potential than older ones. Furthermore, we concluded that STAS was related to an increased tendency for the ki-67 index, which has already been reported by Sun and his colleagues [27]. Above all, further research is indispensable to explore this phenomenon more precisely.

As far as I concerned, evaluating the clinical relevance of this nomogram is reflected in the following points. First, transbronchoscopic lung biopsy or transbronchoscopic ultrasound-guided transbronchial needle aspiration and transthoracic needle aspiration are common methods to obtain pathological results prior to the surgery or other treatments. However, bronchial cytology or small biopsy specimens cannot provide precise information for predicting the presence of STAS [28, 29]. In addition, STAS was observed in the tumor boundary beyond the edge of the main tumor. However, this site might not be punctured. For the above reason, we can develop individualized surgical strategy through our risk prediction nomogram. For example, we can predict the possible status of STAS using this nomogram and patients with STAS positive will accept lobetomy without selecting sublobar resection. Also, the removed specimens from patients receiving neoadjuvant therapy were difficult to be evaluated the presence of STAS. Preoperative prediction model can serve as a predictor for the potential recurrence risk of these cases.Second, the presence of STAS was highly accociated with the expression of PD-L1 in our group. Immunotherapy showed promosing results in the treatment of late stage NSCLC. However, it is not known the potential effect on preventing of recurrence in early stage patients. Prophylactic immunotherapy for the treatment of early stage patients may be validated in large scale clinical trials.

There were several limitations in our analysis. First, its retrospective nature and the relatively small number of patients enrolled in our single center limited the accuracy of our findings. Moreover, further data on PD-L1 expression are necessary to probe the correlation between STAS and PD-L1, which may open an era for immunotherapy. Besides, important imaging parameters were not assessed in our analysis. In addition, a relatively low number of late-stage patients were enrolled. Most late-stage patients only undergo CT-guided or bronchoscopy-guided biopsy, and surgical resection is not indicated. The difference in STAS between paired biopsy and resected specimens in late-stage patients will be assessed in future studies.

In conclusion, we successfully established a nomogram model based on five independent predictors of STAS, which has huge prospects for clinical application.

## Data Availability

The data used to support the findings of this study are available from 10.6084/m9.figshare.21432126.v1.
